# An accurate and affordable test for the rapid diagnosis of sickle cell disease could revolutionize the outlook for affected children born in resource-limited settings

**DOI:** 10.1186/s12916-015-0483-4

**Published:** 2015-09-23

**Authors:** Thomas N. Williams

**Affiliations:** Department of Medicine, Imperial College, St Mary’s Hospital, London, W2 1NY UK; KEMRI/Wellcome Trust Research Programme, PO Box 230, Kilifi, Kenya

**Keywords:** Sickle cell disease, Point of care, Diagnostic test

## Abstract

Each year, at least 280,000 children are born with sickle cell disease (SCD) in resource-limited settings. For cost, logistic and political reasons, the availability of SCD testing is limited in such settings and consequently 50–90 % of affected children die undiagnosed before their fifth birthday. The recent development of a point of care method for the diagnosis of SCD – the Sickle SCAN™ device – could afford such children the prompt access to appropriate services that has transformed the outlook for affected children in resource-rich areas. In research published in *BMC Medicine*, Kanter and colleagues describe a small but carefully conducted study involving 208 children and adults, in which they found that by using Sickle SCAN™ it was possible to diagnose the common forms of SCD with 99 % sensitivity and 99 % specificity, in under 5 minutes. If repeatable both in newborn babies and under real-life conditions, and if marketed at an affordable price, Sickle SCAN™ could revolutionize the survival prospects for children born with SCD in resource-limited areas.

Please see related article: http://dx.doi.org/10.1186/s12916-015-0473-6.

## Background

Sickle cell disease (SCD) is a neglected, chronic, multi-system disorder that is of growing importance in the global health context [1, 2]. SCD is caused by the inheritance of a mutation in the *HBB* gene that results in the production of a structurally abnormal form of β-globin. This variant form of haemoglobin, known as sickle haemoglobin or HbS, polymerizes reversibly under low oxygen tension to alter the shape and rheological properties of red blood cells, a phenomenon that is central to the pathophysiology of SCD [3]. Although SCD is most commonly caused by the homozygous inheritance of HbS (HbSS), it can also result from the coinheritance of HbS with other mutations of the *HBB* gene, most notable among them being a second structural haemoglobin variant, HbC, and β-thalassaemia, a condition characterized by the reduced production of normal β-globin chains [3].

### SCD in resource-limited settings

The sickle mutation has risen to high allele frequencies in many parts of Africa, India and the Middle East because carriers (with HbAS) are strongly protected against death from *Plasmodium falciparum* malaria [4, 5]. As a consequence, more than 90 % of global SCD births – at least 280,000 births each year [6] – occur in resource-limited regions of the world. Nevertheless, in comparison to the minority of affected subjects who are born in resource-rich regions, the outlook for these children is stark. Since the introduction of newborn screening throughout much of Europe and North America, the majority of children born with SCD in these regions are diagnosed early, placed on life-long care, and can expect to live to middle-age and beyond [7]. By contrast, however, poor diagnostic facilities coupled with the low priority afforded to SCD in the health plans of many countries in sub-Saharan Africa (SSA) mean that the majority of children born with the condition on the continent go undiagnosed and die from preventable complications before their fifth birthday [8]. As a consequence, SCD is responsible for an increasing proportion of overall childhood mortality in SSA, reaching 6 % or more in a number of countries within the region [9, 10].

### Current methods for the diagnosis of SCD

Key to tackling the early mortality of children with SCD is prompt diagnosis. This allows for the education of parents about how best to look after their affected children and to recognize important danger signs, and for the prevention of complications through the targeted use of vaccinations, antibiotics and anti-malarial drugs. Such measures, which could be easily afforded by many countries within the region [11, 12], were successful in reducing the high early mortality that was previously seen in other parts of the world, and there is no reason to suspect that the same should not be true if widely implemented in resource-limited settings [10]. However, to date, diagnostic facilities for SCD remain poor throughout many such regions, where all too often they are limited to private facilities and beyond the reach of the majority who would benefit.

A number of different methods can be used to diagnose SCD, of which the most common are haemoglobin electrophoresis, high performance liquid chromatography (HPLC), isoelectric focusing and molecular approaches such as PCR [13]. However, all require well-trained staff working with well-maintained equipment in reasonable laboratory facilities, a reliable supply of power, and systems for the delivery and storage of reagents, the commercial costs of which alone can typically run to $5–10 per test. Moreover, laboratory-based approaches require functional systems for sample transport and the return of results. Although pilot newborn screening studies in a number of resource-limited countries have used such approaches successfully [14, 15], they have rarely been rolled out beyond the confines of time-limited research-based projects. Moreover, these cost and logistic constraints mean that even when health workers suspect the condition in older children, testing for SCD is seldom undertaken. It is in this context that the widespread availability of a rapid and reliable diagnostic test could revolutionize the outlook for children born with SCD in many resource-limited settings.

### The Sickle SCAN™ test

In research published in *BMC Medicine*, Kanter and colleagues show that a new point of care diagnostic device, called Sickle SCAN™, can be used to accurately diagnose the most common forms of SCD (HbSS and HbSC) from a capillary sample of blood, in less than 5 minutes [16]. This seemingly easy-to-use test employs a sandwich format chromatographic immunoassay approach for the qualitative measurement of HbA, HbS and HbC, together with α-globin as a positive control. The test has a similar look and feel to devices that are used for the rapid diagnosis of other conditions, including HIV and malaria, with which most practitioners in resource-limited settings will be familiar. Sickle SCAN™ has a number of potential advantages over traditional laboratory methods. Perhaps most importantly, being so rapid the method could contribute to the immediate management of patients in whom clinicians suspect a diagnosis of SCD, allowing for the real-time communication of results and appropriate referral to specialist services. Moreover, the test requires no electricity and should avoid the cost and added complexity of sample transport and the feedback of results.

### Further work needed

The greatest gains will be delivered if Sickle SCAN™ makes feasible the widespread testing of children as early in life as possible – preferably at birth (if delivered at a health facility) or at first contact with a health professional. However, the high and variable concentration during early life of red cell fetal haemoglobin (HbF) means that it cannot be assumed that Sickle SCAN™ will be equally accurate during this period without further studies. Such studies are currently under way, the results of which are anticipated with considerable interest. Similarly, before its wide endorsement, the accuracy of Sickle SCAN™ needs confirming in larger studies conducted in multiple communities under real-world conditions. Finally, a critical issue regarding the likely utility of Sickle SCAN™ will be cost. While this will obviously be determined through negotiation between the manufacturers and their various target groups, it goes without saying that the more cheaply that Sickle SCAN™ can be made available, the greater its potential impact.

## Conclusions

While Sickle SCAN™ is a welcome development, no genetic test comes without a downside. SCD and its carrier status are still associated with considerable stigma in many affected communities [17] and, as for other chronic conditions, unwelcome consequences can ensue if testing is conducted in the absence of proper support. While pre- and post-test counselling by well-trained individuals should mitigate such consequences to some extent, better education regarding SCD in affected communities and improved advocacy at every level are of equal importance. By facilitating the widespread diagnosis of SCD in resource-limited settings, Sickle SCAN™ could make a valuable contribution to this process.

## Abbreviations

HPLC: High performance liquid chromatography
PCR: Polymerase chain reaction
SCD: Sickle cell disease
SSA: Sub-Saharan Africa
